# D1 Receptor Mediated Dopaminergic Neurotransmission Facilitates Remote Memory of Contextual Fear Conditioning

**DOI:** 10.3389/fnbeh.2022.751053

**Published:** 2022-02-17

**Authors:** Nae Saito, Makoto Itakura, Toshikuni Sasaoka

**Affiliations:** ^1^Department of Comparative and Experimental Medicine, Brain Research Institute, Niigata University, Niigata, Japan; ^2^Department of Molecular and Cellular Medicine, Graduate School of Medical and Dental Sciences, Niigata University, Niigata, Japan; ^3^Department of Biochemistry, Kitasato University School of Medicine, Sagamihara, Japan

**Keywords:** dopamine D1 receptor (D1R), aversive memory, remote memory, c-Fos, SNAP-25

## Abstract

Dopaminergic neurotransmission *via* dopamine D1 receptors (D1Rs) is considered to play an important role not only in reward-based learning but also in aversive learning. The contextual and auditory cued fear conditioning tests involve the processing of classical fear conditioning and evaluates aversive learning memory. It is possible to evaluate aversive learning memory in two different types of neural transmission circuits. In addition, when evaluating the role of dopaminergic neurotransmission *via* D1R, to avoid the effects in D1R-mediated neural circuitry alterations during development, it is important to examine using mice who D1R expression in the mature stage is suppressed. Herein, we investigated the role of dopaminergic neurotransmission *via* D1Rs in aversive memory formation in contextual and auditory cued fear conditioning tests using D1R knockdown (KD) mice, in which the expression of D1Rs could be conditionally and reversibly controlled with doxycycline (Dox) treatment. For aversive memory, we examined memory formation using recent memory 1 day after conditioning, and remote memory 4 weeks after conditioning. Furthermore, immunostaining of the brain tissues of D1RKD mice was performed after aversive footshock stimulation to investigate the distribution of activated c-Fos, an immediate-early gene, in the hippocampus (CA1, CA3, dentate gyrus), striatum, amygdala, and prefrontal cortex during aversive memory formation. After aversive footshock stimulation, immunoblotting was performed using hippocampal, striatal, and amygdalar samples from D1RKD mice to investigate the increase in the amount of c-Fos and phosphorylated SNAP-25 at Ser187 residue. When D1R expression was suppressed using Dox, behavioral experiments revealed impaired contextual fear learning in remote aversion memory following footshock stimulation. Furthermore, expression analysis showed a slight increase in the post-stimulation amount of c-Fos in the hippocampus and striatum, and a significant increase in the amount of phosphorylated SNAP-25 in the hippocampus, striatum, and prefrontal cortex before and after stimulation. These findings indicate that deficiency in D1R-mediated dopaminergic neurotransmission is an important factor in impairing contextual fear memory formation for remote memory.

## Introduction

Dopamine neurotransmission through D1Rs and D1-like receptors is thought to play an important role in aversive memory (El-Ghundi et al., 2001; Ortiz et al., 2010; Sarinana et al., 2014; Soares-Cunha et al., 2016b; Schultz, 2019). On the other hand, dopamine neurotransmission through D2 dopamine receptors (D2Rs) and D2-like receptors is also thought to play an important role in aversive memory (Bromberg-Martin et al., 2010; Danjo et al., 2014; Yamaguchi et al., 2015; Soares-Cunha et al., 2016b; Kawahata et al., 2021).

To date, it has been reported that the direct and indirect pathways of the basal ganglia are composed of medium spiny neurons expressing D1R and D2R, respectively (Gerfen et al., 1990), and that dopamine neurotransmission through D1R mediates behavioral promotion and reward learning, while dopamine neurotransmission through D2R mediates behavioral suppression and aversive learning (Kravitz and Kreitzer, 2012; Volman et al., 2013). However, in recent years, these concepts have been re-evaluated, and the complexity of the basal ganglia circuit has been investigated (Calabresi et al., 2014; Soares-Cunha et al., 2016a,b; Shin et al., 2018).

D1R knockout (KO) mice or genetically modified mice, including conditional D1R knockdown mice using drug administration or the Cre-loxP system, have been utilized to elucidate the role of dopamine transmission through D1R in fear conditioning (El-Ghundi et al., 2001; Ortiz et al., 2010; Ikegami et al., 2014). However, the results of these analyses have been inconsistent, and the role of D1R-mediated dopamine neurotransmission in the hippocampus, amygdala, prefrontal cortex, and striatum remains to be fully elucidated.

In this study, we investigated the effects of D1R suppression on long-term memory of contextual and auditory cued fear conditioning as aversive learning in mature mice, as well as the role of D1Rs in the hippocampus, dorsomedial striatum (DMS), prelimbic region of medial prefrontal cortex (mPFC), and basal amygdala (BA). Fear conditioning memory tests were conducted 1 day after conditioning to examine recent memories and 4 weeks after conditioning to evaluate remote memories in long-term memory to investigate the effect of D1Rs suppression. Specifically, we observed the expression of c-Fos that is involved in the molecular mechanisms of learning and memory and is rapidly expressed during long-term memory formation (Milanovic et al., 1998; Tischmeyer and Grimm, 1999; Fleischmann et al., 2003; Miyashita et al., 2018). Contextual fear memories require the hippocampus, amygdala, striatum, and mPFC (Goshen et al., 2011; Ikegami et al., 2014; Stubbendorff et al., 2019; Mizuno et al., 2020), and auditory cued fear memories require the amygdala or striatum and do not depend on the hippocampus (Phillips and LeDoux, 1992; Pare et al., 2004; Goshen et al., 2011). Different neural circuits are thought to be important for contextual fear conditioning and auditory cued fear conditioning. In this study, we examined the effects of the suppression of D1R expression on fear memory formation in contextual and auditory cued fear conditioning in mature mice and compared the results of this study with results regarding aversive memory formation in the passive avoidance test reported previously (Saito et al., 2020). Furthermore, we analyzed the phosphorylation of synaptosomal-associated protein of 25 kDa (SNAP-25) at Ser187, which is involved in cognitive function and stress (Genoud et al., 1999; Yamamori et al., 2014), and investigated the effects of D1R suppression on aversive memory formation.

## Materials and Methods

### Mice

C57BL/6 mice were purchased from CLEA Japan (Tokyo, Japan). The generation of D1R knockdown (KD) mice (D1R homozygous knockout/Tet/Off system-based compound-transgenic mice) was performed following previously published protocols (Chiken et al., 2015; Okubo et al., 2018; Saito et al., 2020). Only male mice were used in the contextual and cued fear conditioning tests.

Mice were maintained under a 12 h light/dark cycle (lights on at 7:00 AM), with *ad libitum* access to food and water in specific-pathogen-free conditions. All experiments were performed in accordance with the guidelines of the National Institutes of Health and the Ministry of Education, Culture, Sports, Science and Technology (MEXT) of Japan, and in compliance with the protocol that was reviewed by the Institutional Animal Care and Use Committee and approved by the President of Niigata University (Permit Number: SA00954) as previously described (Saito et al., 2020).

### Grouping and Doxycycline (Dox) Treatment

**FIGURE 1:**
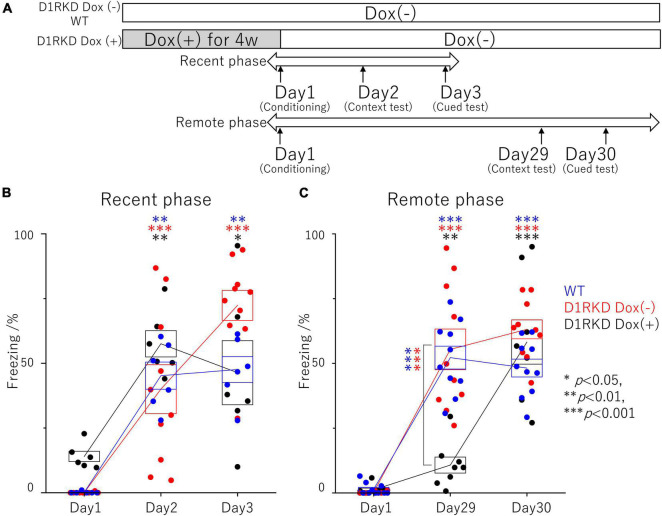
Contextual and auditory cued fear conditioning test. **(A)** Experimental schedule. In the D1RKD Dox (+) group, Dox (2 mg/mL) was administered for 4 weeks prior to the commencement of the experiment and until after the completion of conditioning as the learning session on Day 1, with mice provided drinking water without Dox thereafter. The D1RKD Dox (–) and wild-type (WT) groups were always given only Dox-free water. Conditioning on Day 1, context test on Day 2, and auditory cued test on Day 3 were conducted for recent phase tests in all three groups [WT; *n* = 6, D1RKD Dox (–); *n* = 10, D1RKD Dox (+); *n* = 6]. On the other hand, Conditioning on Day 1, context test on Day 29, and cued test on Day 30 were conducted for remote phase tests in all three groups [WT; *n* = 10, D1RKD Dox (–); *n* = 10, D1RKD Dox (+); *n* = 8]. **(B)** In the recent phase tests, the freezing time of mice was recorded for all three groups during the context tests of Day 2 and the auditory cued tests of Day 3 as the test session, *p < 0.05; ^**^p < 0.01; ^***^p < 0.001. **(C)** In the remote phase tests, the freezing time of mice was recorded for all three groups during the context tests of Day 29 and the auditory cued tests of Day 30 as the learning session. The blue circles indicate the values of WT groups, the red circles indicate the values of D1RKD Dox (–) groups, the black circles indicate the values of D1RKD Dox (+) groups, ^**^p < 0.01; ^***^p < 0.001.

**FIGURE 2:**
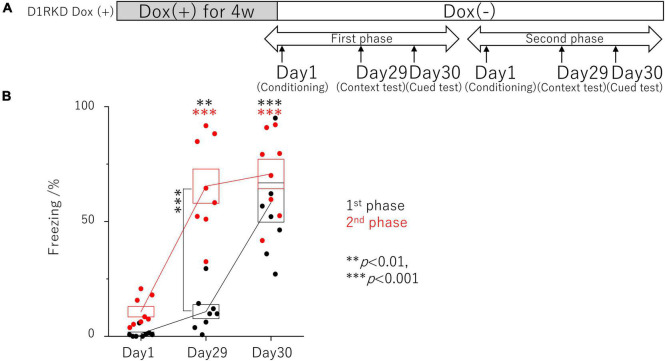
Contextual and auditory cued fear conditioning test with the same mice for remote phase. **(A)** Experimental schedule. Experiments were conducted only in the D1RKD Dox (+) group (*n* = 8) and the experiments of the 1st phase and 2nd phase were performed with the same mice. Dox (2 mg/mL) was administered for 4 weeks prior to the commencement of the experiment and until after the completion of conditioning as the learning session on Day 1, with mice provided drinking water without Dox thereafter. Conditioning on Day 1, contextual test on Day 29, and auditory cued test on Day 30 conducted for 1st phase and 2nd phase tests, respectively. **(B)** In 1st phase and 2nd phase, the freezing time of mice was recorded during the contextual tests of Day 29, and the auditory cued tests of Day 30 as the test session with the same mice, respectively. The black circles indicate the values in 1st phase, and the red circles indicate the values in 2nd phase, ^**^p < 0.01; ^***^p < 0.001.

In D1RKD mice, 2.0 mg/mL doxycycline (Dox; Sigma Aldrich, United States) was used to knock down D1R expression, as previously described (Chiken et al., 2015; Okubo et al., 2018; Saito et al., 2020). For mice treated with Dox, Dox was administered *via* drinking water containing 5% sucrose for 4 weeks prior to behavioral tests and until the completion of the conditioning experiment (see section “Contextual and Auditory Cued Fear Conditioning Test,” Figures 1A, 2A), after which all mice received drinking water without Dox for the remainder period. Mice that were not treated with Dox received Dox-free water for the entire duration of the experiment. After termination of Dox administration, the expression level of D1Rs recovered to the same level as that before Dox administration (Chiken et al., 2015).

In the contextual and auditory cued fear conditioning test, D1RKD mice were assigned to D1RKD Dox (−) and D1RKD Dox (+) groups. D1RKD Dox (−) mice were used with ten mice per group. D1RKD Dox (+) were used with six and eight mice in recent and remote phase tests, respectively; six and ten wild-type (WT) mice were used as the control group in recent and remote phase tests, respectively.

In the immunohistochemical analysis, D1RKD mice were assigned to the following four groups: D1RKD Dox (−) Stimulation (−) (*n* = 3), D1RKD Dox (−) Stimulation (+) (*n* = 3), D1RKD Dox (+) Stimulation (−) (*n* = 3), and D1RKD Dox (+) Stimulation (+) (*n* = 3); two groups of WT mice, Stimulation (−) (*n* = 3) and Stimulation (+) (*n* = 3), were used as the control groups.

In the immunoblot analysis, D1RKD mice were assigned to the following six groups: D1RKD Dox (−) Stimulation (−) (*n* = 3), D1RKD Dox (−) Stimulation (+) (*n* = 3), D1RKD Dox (+) Stimulation (−) (*n* = 3), D1RKD Dox (+) Stimulation (+) (*n* = 3), D1RKD Dox (+→−) Stimulation (−) (*n* = 3), D1RKD Dox (+→−) Stimulation (+) (*n* = 3); two groups of WT mice, Stimulation (−) (*n* = 3) and Stimulation (+) (*n* = 3), were used as control groups.

### Contextual and Auditory Cued Fear Conditioning Test

A computer-controlled fear conditioning system was used for contextual and auditory cued fear conditioning tests (O’Hara & Co., Japan). A clear chamber (16 × 14 × 10 cm) for conditioning and contextual tests was set in a sound-insulating box (30 lux) with white walls. A white chamber (17 × 10 × 10 cm) for auditory cued tests was placed in a sound-insulating box (30 lux) with black walls. Mice were subjected to a conditioning session for 5 min on Day 1, a contextual test session for 5 min on Day 2 or Day 29, and an auditory cued test session for 5 min on Day 3 or Day 30.

In the conditioning session, a tone (65 dB, white noise) was presented for 20 s, and a mild footshock (0.3 mA, 2 s) was presented in the last 2 s through a floor grid. This process was performed three times for 5 min each. The mice were returned to their home cages 60 s after shock termination. Before the initial tone and footshock presentation and in the contextual test session, mice were placed back into a clear chamber used in the conditioning session for 5 min without footshock or tone stimulus. In the auditory cued test session, the mice were placed in a white chamber. A tone stimulus was presented for 2 min after 2 min of placing the mice in the white chamber. The behaviors of the mice were monitored using a CCD camera, and freezing behaviors were analyzed using Time FZ2 software (O’Hara & Co., Tokyo, Japan).

### Treatment Prior to Histochemistry and Immunoblot

Footshock stimulation was carried out in a fear conditioning system or a step-through-type apparatus, comprising light and dark compartments separated by a removable door for the passive avoidance test (O’Hara & Co., Japan). In the fear conditioning system, each mouse was placed in the same way as in the fear conditioning tests. In the passive avoidance apparatus, each mouse was placed in the dark compartment and received a 2 s 0.3 mA electric footshock. Brain samples were collected 1 h after the footshock stimulus was delivered.

### Histochemistry and Cell Counting

D1RKD mice and WT mice were anesthetized with a mixture of medetomidine hydrochloride [0.75 mg/kg body weight (BW)], midazolam (4 mg/kg BW), and butorphanol tartrate (5 mg/kg BW). The mice were then perfused with phosphate-buffered saline (PBS, pH 7.4) and 4% paraformaldehyde in 0.1 M phosphate buffer (PB, pH 7.4) through the left ventricle. The brains were removed, post-fixed overnight in 4% paraformaldehyde in 0.1 M PB (pH 7.4), and equilibrated overnight with 30% sucrose in 0.1 M PB (pH 7.4) at 4°C. The brains were embedded in OCT compound (Sakura Finetek, Japan), frozen over isopropanol (FUJIFILM Wako Pure Chemical Corporation, Japan) in liquid nitrogen, and cut on a cryostat CM1950 (Leica, Germany) into 40 μm thick coronal sections. Free-floating sections were treated with 3% H_2_O_2_ and 10% methanol in PB (pH 7.4) for 10 min and then blocked with 5% Blocking One (Nacalai Tesque) in 0.1% Triton in PBS (PBST) for 1 h after washing with PBS. Sections were incubated at 4°C with a polyclonal rabbit anti c-Fos antibody (1: 600, Cell Signaling) in blocking solution overnight, washed with PBST, and then incubated with biotinylated goat anti rabbit IgG (1: 300, Jackson Laboratories, United States) for 2 h. Thereafter, sections were incubated for 1 h with Vectastain Elite ABC Kit (Vector Laboratories, United States), washed with PBST, and then incubated with SIGMAFAST 3,3’-diaminobenzidine (DAB) (Sigma Aldrich, United States). After washing with distilled water, sections were mounted on glass slides, counterstained with 0.5% methyl green (FUJIFILM Wako Pure Chemical Corporation, Japan), and washed with distilled water. Finally, immunostained sections on mounted slides were fixed by serial dehydration in alcohol and lemosol and mounted using Softmount (FUJIFILM Wako Pure Chemical Corporation, Japan). Images of immunostained sections were acquired using a BZ-9000 microscope (Keyence, Japan) with BZ-II Analyzer software (Keyence, Japan). Immunoreactive cells were counted bilaterally in three sections, each from three mice by an experimenter blinded to the treatment conditions.

**FIGURE 3:**
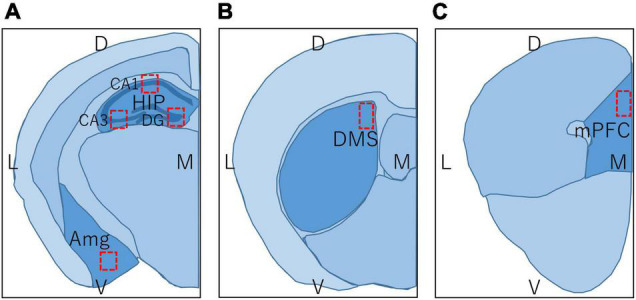
Regions in the brain of mice used in immunohistochemistry and immunoblot. L, lateral; M, medial; D, dorsal; V, ventral; HIP, hippocampus; CA1, hippocampal CA1 region; CA3, hippocampal CA3 region; DG, dentate gyrus; Amg, amygdala; DMS, dorsomedial striatum; mPFC, medial prefrontal cortex. Brain section map of the hippocampus and amygdala (**A**; bregma –1.34 to –2.06 mm), DMS (**B**; bregma –0.10 to 0.98 mm), and prelimbic region of mPFC (**C**; bregma 1.70 to 1.98 mm). Rectangles in **(A–C)** represent the areas in which the number of c-Fos positive cells was counted.

Regions in the brain of mice examined by immunohistochemistry are shown in Figure 3, and c-Fos positive cells were counted within the red rectangles. Brain sections including hippocampus and amygdala (Figure 3A; bregma −1.34 to −2.06 mm), DMS (Figure 3B; bregma −0.10 to 0.98 mm), and prelimbic region of mPFC (Figure 3C; bregma 1.70 to 1.98 mm) were analyzed. To examine each region of the hippocampus in detail, the number of c-Fos positive cells within the rectangles (300 × 300 μm) in the CA1, CA3, and dentate gyrus (DG) regions were counted separately. The number of c-Fos positive cells in the BA, DMS, and mPFC were counted within the rectangles (500 × 500 μm, 300 × 300 μm, and 300 × 300 μm, respectively), as shown in Figures 3A–C, respectively.

### Immunoblotting

D1RKD mice and WT mice were sacrificed by cervical dislocation, and their brains were removed. The brains were sliced coronally into 1 mm thick sections. Regions in the brains of mice used for immunoblotting are shown in Figure 3. However, in contrast to immunohistochemistry, for immunoblotting, the entire hippocampus was used, including both the dorsal and ventral sides (Figure 3A). In addition, the entire striatum, including the DMS and the entire amygdala, including the BA was used (Figures 3A,B). Hippocampal tissues containing CA1, CA3, and DG were collected from the sections of three mice. Striatum and amygdala tissues were obtained from the sections of three mice. Each tissue was homogenized in phase-transfer surfactant buffer (PTS, 12 mM sodium deoxycholate, 12 mM sodium N-dodecanoylsarcosinate, and 200 mM triethylammonium bicarbonate) with cOmplete EDTA-free protease inhibitor (Sigma Aldrich, United Kingdom), PhosStop (Sigma Aldrich, United Kingdom), and 1 mM EDTA. Homogenized proteins were quantified using a BCA kit (Wako, Japan). Homogenates were solubilized in sample buffer (125 mM Tris–HCl, pH 7.4, 3.3% glycerol, 2% SDS, and 50 mM DTT) and heated at 90°C for 5 min. The proteins were then separated on 5 – 20% polyacrylamide gel (ATTO, Japan) loaded at 5 μg in SDS sample buffer at 15 mA for 90 min. These gels were blotted onto PVDF membranes (Immobilon-P, Merck Millipore) in blotting buffer (192 mM glycine, 0.037% SDS, 20% methanol, 100 mM Tris–HCl, pH 8.8) at 20 V for 60 min, and then blocked with 5% non-fat milk in TBST (150 mM NaCl, 0.2% Tween 20, 20 mM Tris–HCl, pH 7.5) for 30 min at room temperature. These membranes were incubated with rabbit anti c-Fos antibody (1: 1,000, Cell Signaling), mouse anti actin antibody (1: 1,000, BD Biosciences, United States), rabbit anti SNAP-25 antibody (1 μg/ml) (Yamamori et al., 2011), or rabbit anti Phospho-SNAP-25 (Ser187) antibody (1: 500) (Iida et al., 2013) in 1% non-fat milk with TBST at 4°C overnight, washed with TBST, and then incubated with peroxidase-conjugated donkey anti-rabbit IgG (H + L) (1: 2,000, Jackson Laboratories, United States) or peroxidase-conjugated donkey anti-mouse IgG (H + L) (1: 1,000, Jackson Laboratories, United States) for 1 h. Immunoreacted samples were visualized using a chemiluminescent reagent (Chemi-Lumi One, Nacalai Tesque or Immobilon Western Chemiluminescent HRP, Sigma Aldrich), and images were acquired using a luminescence image analyzer (LAS 4000, GE Healthcare, Sweden) with ImageQuant TL software (GE Healthcare, Sweden).

### Statistical Analysis

All data were analyzed using Origin 2021 (OriginLab, United States). Data from the contextual and auditory cued fear conditioning tests and cell counting data from immunohistochemistry were analyzed using the Mann-Whitney test. Immunoblot data were analyzed using the two sample *t* test. Significance was set at *p* < 0.05.

## Results

### Contextual and Auditory Cued Fear Conditioning Test

The effect of suppressing D1R expression on fear memory formation was examined using a contextual and auditory cued fear conditioning test in the recent and remote phase (Figure 1A). First, recent phase examinations found significantly longer freezing responses in both the context test (Day 2) and auditory cued test (Day 3) than those before conditioning. There was no significant difference between the three groups on both the context test (Day 2) and auditory cued test (Day 3) (Figure 1B). Next, the remote phase contextual and auditory cued fear conditioning tests were conducted using different mice from those used in the recent phase tests. In the remote phase context tests (Day 29), D1RKD Dox (+) mice showed almost no freezing response, similar to before conditioning (Day 1). The freezing responses of D1RKD Dox (+) mice were clearly different from those of the other two groups (D1RKD Dox (−) and WT), while the freezing responses in the remote phase context tests (Day 29) of D1RKD Dox (−) and WT mice were increased and similar to those seen in the recent phase context tests (Day 2). On the other hand, D1RKD Dox (+) mice showed a significantly increased percentage of time spent freezing in the auditory cued tests (Day 30), which was equivalent to WT and D1RKD Dox (−) mice, albeit with some amount of variation (Figure 1C). Furthermore, D1RKD Dox (−) mice overexpressing D1Rs showed a significantly increased percentage of time spent freezing compared to WT mice in the auditory cued tests [Day 3 (*p* < 0.05), Day 30 (*p* < 0.05)].

In addition, the effects of recovery of D1R expression were examined using the same mice as those in the remote phase contextual and auditory cued fear conditioning tests (Figure 2A). In the first test phase in the context tests (Day 29), D1RKD Dox (+) mice showed almost no freezing response, similar to before conditioning (Day 1) (Figure 2B). In the second test phase, following footshock conditioning on Day 1, D1RKD Dox (+) mice showed a significantly increased percentage of freezing on the context tests (Day 29). On the other hand, in the auditory cued fear conditioning test, both D1RKD mice with suppressed D1R expression in the first test phase and the same mice with recovering D1R expression in the second phase showed freezing behavior. These results using the same D1RKD mice were consistent with those obtained using different D1RKD mice (Figure 2A).

### Immunohistochemistry

**FIGURE 4:**
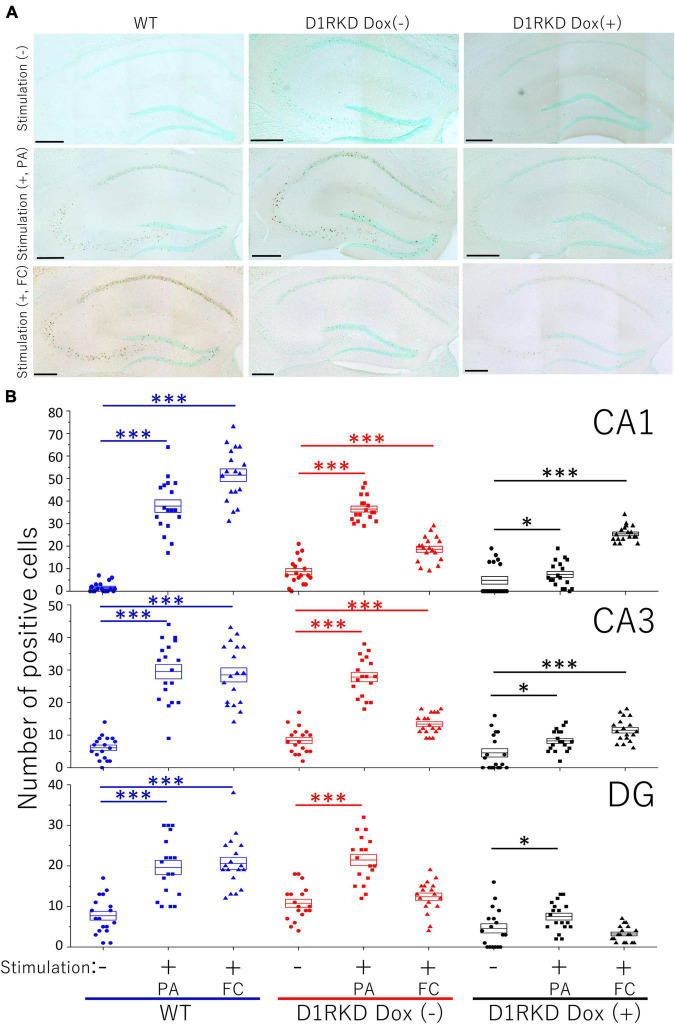
Immunohistochemical analysis of c-Fos expression in the hippocampus (CA1, CA3, and DG) after 1 h of electric footshock stimulus using a step-through-type apparatus for the passive avoidance test (PA) or fear conditioning system (FC). **(A)** Representative immunohistochemical staining of c-Fos positive cells. Scale bar, 300 μm. **(B)** The number of positive cells in CA1, CA3, DG in immunohistochemistry. All groups, *n* = 18. The blue color indicates the values of WT groups, the red color indicates the values of D1RKD Dox (–) groups, and the black color indicates the values of D1RKD Dox (+) groups. The circle, square, and triangle indicate no stimulation, stimulation by PA, and stimulation by FC, respectively, *p < 0.05; ^***^p < 0.001.

**FIGURE 5:**
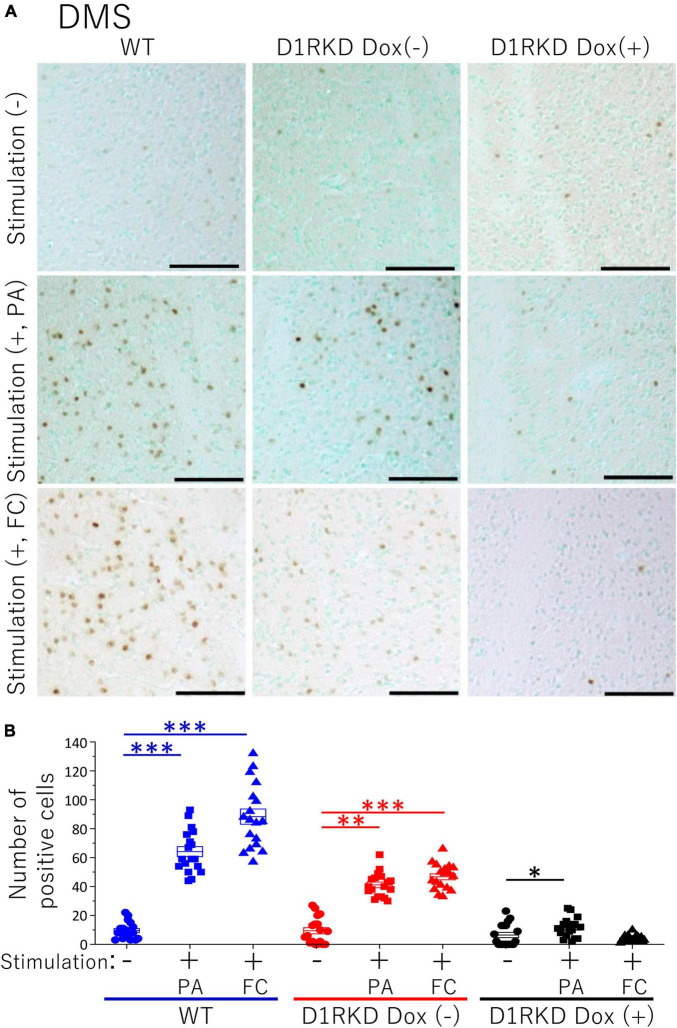
Immunohistochemical analysis of c-Fos expression in DMS after 1 h of electric foot shock stimulus using a step-through-type apparatus for the passive avoidance test (PA) or fear conditioning system (FC). **(A)** Representative immunohistochemical staining of c-Fos positive cells. Scale bar, 100 μm. **(B)** The number of positive cells in DMS in immunohistochemistry. All groups, *n* = 18. The blue color indicates the values of WT groups, the red color indicates the values of D1RKD Dox (–) groups, and the black color indicates the values of D1RKD Dox (+) groups. The circle, square, and triangle indicate no stimulation, stimulation by PA, and stimulation by FC, respectively, *p < 0.05; ^**^p < 0.01; ^***^p < 0.001.

**FIGURE 6:**
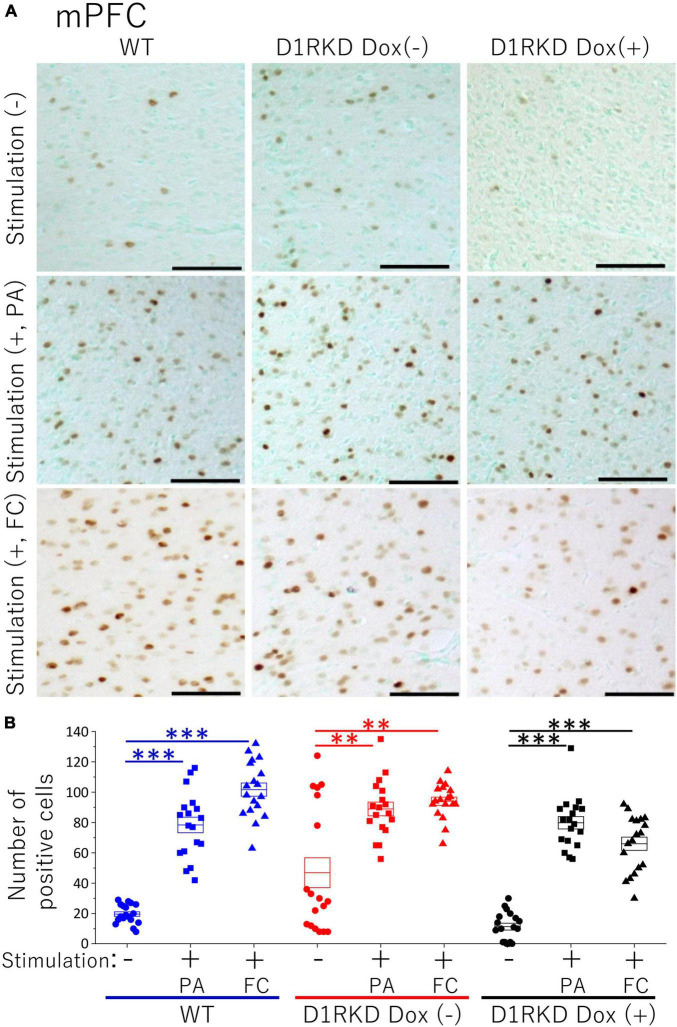
Immunohistochemical analysis of c-Fos expression in mPFC after 1 h of electric footshock stimulus using a step-through-type apparatus for the passive avoidance test (PA) or fear conditioning system (FC). **(A)** Representative immunohistochemical staining of c-Fos positive cells. Scale bar, 100 μm. **(B)** The number of positive cells in mPFC in immunohistochemistry. All groups, *n* = 18. The blue color indicates the values of WT groups, the red color indicates the values of D1RKD Dox (–) groups, and the black color indicates the values of D1RKD Dox (+) groups. The circle, square, and triangle indicate no stimulation, stimulation by PA, and stimulation by FC, respectively, ^**^p < 0.01; ^***^p < 0.001.

**FIGURE 7:**
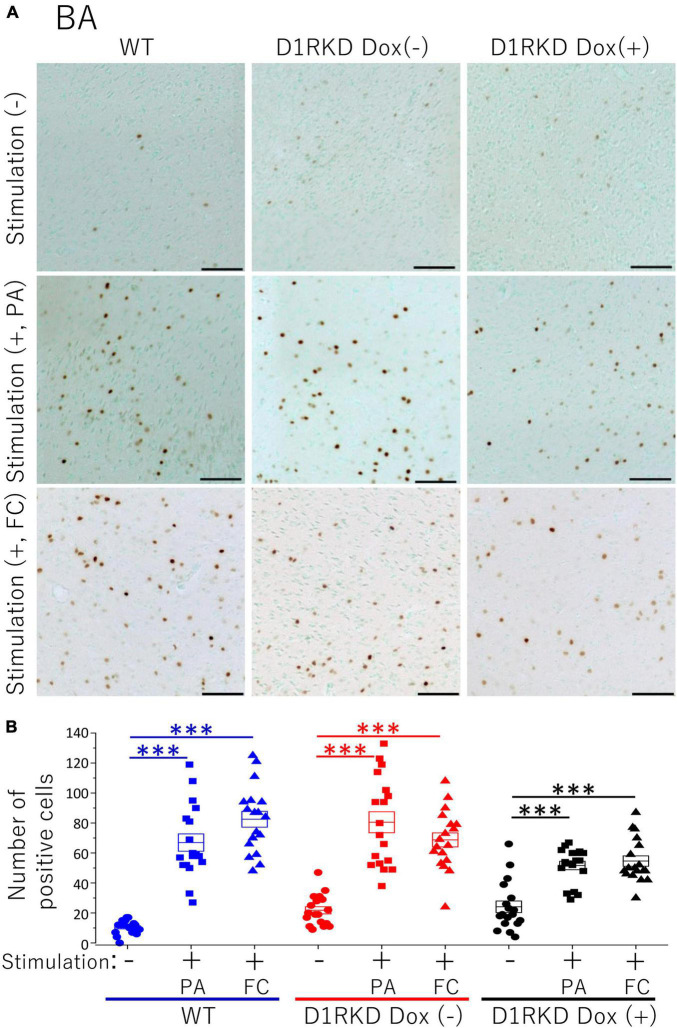
Immunohistochemical analysis of c-Fos expression in BA after 1 h of electric footshock stimulus using a step-through-type apparatus for the passive avoidance test (PA) or fear conditioning system (FC). **(A)** Representative immunohistochemical staining of c-Fos positive cells. Scale bar, 100 μm. **(B)** The number of positive cells in BA in immunohistochemistry. All groups, *n* = 18. The blue color indicates the values of WT groups, the red color indicates the values of D1RKD Dox (–) groups, and the black color indicates the values of D1RKD Dox (+) groups. The circle, square, and triangle indicate no stimulation, stimulation by PA, and stimulation by FC, respectively, ^***^p < 0.001.

Representative immunohistochemical staining images of the hippocampus are shown in Figure 4A. Hippocampal CA1, CA3, and DG regions in Figure 4 were enlarged and shown in Supplementary Figures 1A–C, respectively. Distributions of c-Fos positive cells in the hippocampus of WT and D1RKD Dox (−) mice with footshock stimulation by a fear conditioning (FC) or a passive avoidance (PA) apparatus were compared with those of the WT and D1RKD Dox (−) mice without stimulation. In the case of PA footshock stimulation, there was an increase in the number of c-Fos positive cells in a large area of the hippocampi of WT and D1RKD Dox (−) mice with footshock stimulation. In contrast, c-Fos positive cells were hardly observed in either case of D1RKD Dox (+) mice with or without stimulation (Figure 4A). In the case of FC footshock stimulation, the number of c-Fos-positive cells increased in a large area of the hippocampi of footshock-stimulated WT mice. In contrast, c-Fos-positive cells were not often observed in either D1RKD Dox (−) and D1RKD Dox (+) mice, with or without stimulation (Figure 4A). In both cases of PA and FC footshock stimulation, when the number of c-Fos positive cells was counted in all the CA1, CA3, and DG regions, there was a significant increase in the two groups (WT and D1RKD Dox (−) mice) when comparing the stimulated and unstimulated groups. However, the number of positive cells D1RKD Dox (−) mice was lower in FC than in PA. Moreover, in the case of PA apparatus, there was a little increase in the hippocampal CA1, CA3, and DG regions of the D1RKD Dox (+) mice group when comparing the stimulated and unstimulated groups (Figure 4B). In the case of FC footshock stimulation, the number of positive cells in the hippocampal CA1 and CA3 regions was slightly increased, but that in the DG region was almost unchanged (Figure 4B). In addition, the distributions of c-Fos positive cells in the DMS of WT, D1RKD Dox (−), and D1RKD Dox (+) mice with and without stimulation were found to be similar to those observed in the hippocampus as described above (Figure 5A). When the number of c-Fos positive cells was counted in DMS, there was a significant increase in the both groups (WT and D1RKD Dox (−) mice) when comparing the stimulated and unstimulated groups. However, there was a little increase in the DMS or hippocampus of the D1RKD Dox (+) mice group when comparing the stimulated and unstimulated groups (Figure 5B). In contrast, an apparent increase in the number of c-Fos positive cells was observed in the mPFC and BA in the mice of the three groups [WT, D1RKD Dox (−), and D1RKD Dox (+)] that received stimulation (Figures 6A, 7A). Furthermore, the number of c-Fos positive cells in mice with stimulation was significantly different from that in mice without stimulation in the three groups (*p* < 0.001) (Figures 6B, 7B).

### Immunoblotting

**FIGURE 8:**
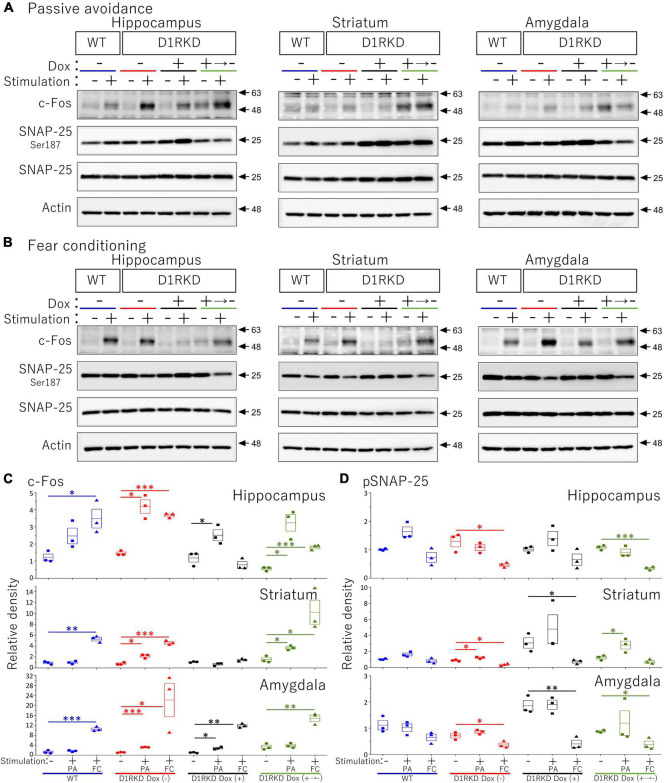
Immunoblot analysis of c-Fos, SNAP-25 phosphorylation at Ser187, SNAP-25, and Actin expression in homogenates from the hippocampus, striatum, and amygdala after 1 h of electric footshock stimulus using a step-through-type apparatus for the passive avoidance test (PA) or fear conditioning system (FC). **(A)** Representative immunoblot images using the apparatus for PA. **(B)** Representative immunoblot images using the system for FC. Samples without electric footshock stimulus were used as controls. Actin was used as a loading control. **(C)** The relative band intensities of c-Fos. **(D)** The relative band intensities of SNAP-25 phosphorylation at Ser187. These band intensities were represented as the ratio of WT mice without stimulation as control of two type stimulation, separately. Actin was used as a loading control. Data shown are from three independent experiments. The blue color indicates the values of WT groups, the red color indicates the values of D1RKD Dox (–) groups, the black color indicates the values of D1RKD Dox (+) groups, and the green color indicates the values of D1RKD Dox (+→–). The circle, square, and triangle indicate no stimulation, stimulation by PA, and stimulation by FC, respectively. All groups, *n* = 3, *p < 0.05; ^**^p < 0.01; ^***^p < 0.001.

Representative immunoblotting images are shown in Figure 8. First, in the case of FC footshock stimulation, c-Fos protein levels were greatly increased in both groups (WT, D1RKD Dox (−) mice), but the increase in c-Fos protein levels in D1RKD Dox (+) mice was quite small compared with those in WT and D1RKD Dox (−) mice in the hippocampus and striatum. Second, in the case of PA footshock stimulation, c-Fos protein levels were significantly increased in D1RKD Dox (−) mice, but those in D1RKD Dox (+) mice were relatively small (Figure 8C). These results were consistent with those of immunohistochemical analysis, except for the increase in the hippocampal CA1 region in the case of FC apparatus. Furthermore, c-Fos protein levels were also examined in D1RKD Dox (+→−) mice in which D1R expression was restored after termination of Dox administration. c-Fos protein levels tended to be similar to those in D1RKD Dox (−) mice before Dox administration. This result indicates that the restoration of D1R expression following suppression led to the recovery of the response to stimulation with respect to the induction of c-Fos expression.

In contrast, in the amygdala, the amount of c-Fos protein was greatly increased by stimulation in all four groups (WT, D1RKD Dox (−), D1RKD Dox (+), and D1RKD Dox (+→−) mice), and also these results were consistent with the results of immunohistochemical analysis, in the case of FC footshock stimulation. In the case of PA footshock stimulation, c-Fos protein levels increased slightly more than in the case of FC footshock stimulation, but the same tendency as that of FC stimulation was observed (Figure 8C). The amount of SNAP-25 protein was approximately the same in all four groups (WT, D1RKD Dox (−), D1RKD Dox (+), and D1RKD Dox (+→−) mice).

Next, in the case of FC footschock stimulation, the amount of phosphorylated SNAP-25 at Ser187 tended to decrease with stimulation in D1RKD Dox (−), D1RKD Dox (+→−), and D1RKD Dox (+) mice in all observed regions of the hippocampus, striatum, and amygdala. In addition, in the case of PA footshock stimulation, the amount of phosphorylated SNAP-25 at Ser187 significantly increased in D1RKD Dox (−), D1RKD Dox (+→−) mice in region of the striatum following stimulation. In contrast, in the hippocampus and amygdala, there were no significant changes. Interestingly, there was a large increase in the amount of phosphorylated SNAP-25 at Ser187 not only after stimulation but also before stimulation of the D1RKD Dox (+) mice compared to that in WT mice without stimulation. This result was prominent in regions of the striatum and amygdala (Figures 8A,B,D).

## Discussion

To date, D1R knockout (KO) mice or genetically modified mice, including conditional D1R knockout (cKO) mice using the Cre-loxP system have been utilized to elucidate the role of dopamine transmission through D1Rs in fear conditioning (El-Ghundi et al., 2001; Ortiz et al., 2010; Ikegami et al., 2014). However, the results of these analyses have been inconsistent, and the role of D1R-mediated dopamine neurotransmission in fear memory formation in the hippocampus, amygdala, prefrontal cortex, and striatum remains to be fully elucidated. In D1R KO mice, D1R is deficient from the beginning of development, and compensatory mechanisms may have been affected. In the case of drug-induced suppression of D1R function, the effects may differ depending on the timing and method of administration and specificity of the drug to D1R. In addition, in the case of conditional knockout mice using Cre-loxP recombination, the timing of the induction of D1R deletion has a significant effect. Although few of these causes have been reported (Stiedl et al., 1999; Soares-Cunha et al., 2016b; Stubbendorff and Stevenson, 2020), it is considered that one of the causes is the timing of D1R elimination.

We have previously reported that D1R KO mice and D1RKD exhibited different impairments in motor function (Okubo et al., 2018). In these D1RKD mice, D1R expression can be reversibly regulated from birth by administering Dox using the TET-OFF system (Chiken et al., 2015). Despite the same D1R deficiency, these mice showed significant differences in motor function depending on the timing of D1R suppression. First, we compared D1R KO mice, which are deficient in D1R from early development, with D1RKD mice, which underwent D1R suppression immediately after birth to examine motor function in early childhood. The basal motor activity of D1R KO mice was higher than that of WT and D1RKD mice, and no effect of D1R suppression on the basal motor activity was observed in D1RKD mice. In adulthood, the motor activity of D1R-deficient D1RKD mice was lower than that of D1R KO, D1R-expressing D1RKD, and WT mice. On the other hand, the motor coordination of D1R-suppressed D1RKD mice was lower in childhood and adulthood than in WT and D1R KO mice. Although it is unclear which neural circuitry affects the behavioral phenotype, the prenatal stage is important for establishing the basis of motor function, suggesting that the role of dopamine neurotransmission *via* D1Rs is also in a developmental stage during growth. Therefore, the brain may develop compensatory mechanisms to avoid a functional decline in motor function due to D1Rs suppression, and it is possible that a similar phenomenon occurs in fear conditioning. This study used these D1RKD mice, which allows D1R to be reversibly regulated from birth by administering Dox to focus on aversive memory formation during maturity and investigate the effects of D1R suppression.

In the present study, we found the following characteristics of the effect of dopamine neurotransmission *via* D1R on contextual fear memory and auditory cued fear memory. First, D1R suppression had no effect on test results for either recent (Day 3) or remote (Day 30) memories in the auditory cued fear conditioning test. The fact that the expression level of c-Fos in the amygdala was not affected by D1R suppression may be one of the causes. Dopamine neurons affect auditory cued fear memory (Jo et al., 2018); therefore, neurotransmission *via* D2Rs instead of D1Rs may be involved. The amount of phosphorylated SNAP-25 was increased in the amygdala before footshock stimulation, but this may not be involved in the amygdala-mediated neurotransmission pathway following electric footshock. This result suggests that suppression of dopaminergic neurotransmission *via* D1R had minor if any effect on auditory cued fear memory formation. In contrast, overexpression of D1Rs had enhanced effects on recent (Day 3) or remote (Day 30) memories in the auditory cued fear conditioning test. This suggests that D1R-mediated dopaminergic neurotransmission may have a positive effect on the auditory cued fear memory. The alteration of c-Fos expression showed no effect of D1R overexpression, but the amount of phosphorylated SNAP-25 at Ser187 was significantly reduced by stimulation compared to WT mice. The expression of phosphorylated SNAP-25 at Ser187 has been reported to increase with stress (Yamamori et al., 2014), which is contrary to the predicted results. Therefore, elucidating the cause of this effect is a matter for future investigation. Second, although there was no effect of D1R suppression on the test results in contextual fear conditioning for recent memories (Day 2), there was a decrease in performance due to D1R suppression for remote memories (Day 29). In contextual fear conditioning, which is considered to be a hippocampal-dependent form of memory, the performance in the test of recent memory did not decrease despite the no increase in hippocampal c-Fos expression as the immunoblotting results, which is an unexpected result and unlike previously reported findings (Saito et al., 2020). However, when the hippocampus was observed in more detail by immunohistochemistry, the expression of c-Fos increased in the CA1 and CA3 regions, though not as much as in WT mice. In particular, the increase of the c-Fos expression in the CA1 region was significantly higher than in PA footshock stimulation. These results may have affected contextual fear conditioning for recent memories. In addition, recent memory in contextual fear conditioning has been reported to involve not only the hippocampus but also the striatum (Ikegami et al., 2014), amygdala, and mPFC. As shown in Figures 6, 7, c-Fos expression was increased in the BA and mPFC in D1R-mediated neurotransmission deficits. In this study these results may not have affected contextual fear conditioning for recent memories by D1Rs suppression, as similar results were obtained with FC and PA footshock stimulation. Third, the performance based on remote memory in contextual fear conditioning was clearly reduced in the D1R-mediated neurotransmission-deficient state when compared to performance in control mice and was reversible such that the performance became equivalent to that of control mice when the D1R-mediated neurotransmission was restored (Figures 1, 2). This is consistent with the fact that the expression of c-Fos in the hippocampus and striatum, and its upregulation by footshock stimulation, is reversible, and that it is reduced in the D1R-mediated neurotransmission-deficient state and becomes comparable to that in control mice when D1R-mediated neurotransmission is restored (Figures 1, 2, 4, 5, 8). As reported by previous studies (Saito et al., 2020), our results suggest that D1R-mediated neurotransmission in the hippocampus and striatum is important for the formation of remote memories in contextual fear conditioning.

In addition, although it was predicted that the expression level of SNAP-25 phosphorylation would decrease, similar to that of c-Fos due to D1R suppression it was found that SNAP-25 phosphorylation was unexpectedly elevated following D1R suppression compared to that in WT mice. Increased stress induces SNAP-25 phosphorylation (Yamamori et al., 2014). A form of plasticity that induces long-term potentiation involved in learning and memory mechanisms results in the phosphorylation of SNAP-25 (Genoud et al., 1999). However, in this study, D1R-suppressed mice had increased SNAP-25 phosphorylation levels in all observed regions, even before electrical stimulation. Phosphorylated SNAP-25 is a multifunctional protein that plays a role in several processes, such as neurite extension, regulation of ion channel function, and regulation of neurotransmitter release (Osen-Sand et al., 1993; Pozzi et al., 2008). Therefore, this may be mediated by a mechanism independent of the function of stress-related neurons (Yamamori et al., 2014). Furthermore, transient overexpression of SNAP-25 phosphorylation at Ser187 is responsible for its negative effects on calcium dynamics and provides a negative feedback mechanism through the inhibition of voltage-gated calcium channels (Pozzi et al., 2008). Changes in SNAP-25 activity are associated with cognitive deficits found in several disorders such as attention deficit hyperactivity disorder and schizophrenia (Mill et al., 2004; Gosso et al., 2006). Knockdown of SNAP-25 does not affect auditory cued fear memory but reduces long-term memory performance in context fear conditioning (Hou et al., 2004). In contrast, overexpression of SNAP-25 in the dorsal hippocampus reduced the performance of context fear conditioning (McKee et al., 2010). Furthermore, there was a significant decrease in SNAP-25 after 12 h of passive avoidance training (O’Sullivan et al., 2007). Therefore, SNAP-25 expression during memory formation must be tightly regulated, and excessive deviations from normal expression levels are thought to affect cognitive function (McKee et al., 2010). However, these have not been investigated for SNAP-25 phosphorylation at Ser187, and they did not mention whether the amount of phosphorylation affects cognitive function. Considering the effect of SNAP-25 on long-term memory in the hippocampus, the decrease in the amount of phosphorylated SNAP-25 before a new stimulus after the elevated amount before the previous stimulus and the increase in phosphorylated SNAP-25 after a new stimulus are considered to be important for memory formation. In the present study, we found that D1R suppression increased the amount of SNAP-25 phosphorylated at Ser187 before footshock stimulation, and when D1R expression was restored, the amount of SNAP-25 phosphorylated at Ser187 decreased to the same level as that in controls. This may be a result of the prevented downregulation of phosphorylated SNAP-25 from the previous stimulation-induced increase in the amount of phosphorylated SNAP-25 during memory formation, leading to reduced long-term memory in the contextual fear condition. The lack of post-stimulation increase in phosphorylated SNAP-25 due to overexpression of phosphorylated SNAP-25 before stimulation may also contribute to memory decline. SNAP-25 phosphorylation at Ser187 occurs *via* protein kinase C (PKC) (Shimazaki et al., 1996; Nagy et al., 2002). Therefore, investigating the relationship between SNAP-25 phosphorylation at Ser187, D1R, and PKC in long-term memory is a subject for future research.

In our previous study, we reported that in the passive avoidance test where mice were conditioned with the same intensity of footshock as in this study, they showed impairments in both recent and remote memories (Saito et al., 2020). In addition, the D1R-mediated neurotransmission deficit had less effect on the suppression of Arc expression in the hippocampus and a greater effect on the suppression of Arc expression in the cerebral cortex. Unlike the contextual fear conditioning test, the passive avoidance test, which uses the same footshock, may have affected the recent memory because it depends not only on the hippocampus but also on multiple regions such as the striatum, amygdala, and cortices (Lorenzini et al., 1996; Pittenger et al., 2006; Ortiz et al., 2010; Yao et al., 2021). In the contextual and auditory cued fear memories examined in this study, mice were conditioned using three repetitions of the same intensity of footshock, and their recent memory was comparable to that of control mice. However, their remote memory of contextual fear conditioning was impaired. The mice performed as well as control mice in the recent memory task but were impaired in the remote memory task following fear conditioning. These results suggest that the effect of D1R-mediated neurotransmission on the formation of recent context fear memories may depend on the upregulation of c-Fos expression by footshock stimulation, especially in the hippocampal CA1 and CA3 regions, or on the upregulation of c-Fos expression by footshock stimulation in other regions such as the striatum, amygdala, and mPFC, or both. Furthermore, D1R-mediated neurotransmission appears to be involved from the formation to the fixation of remote memory.

## Data Availability Statement

The raw data supporting the conclusions of this article will be made available by the authors, without undue reservation.

## Ethics Statement

The animal study was reviewed and approved by the Institutional Animal Care and Use Committee, Niigata University, Japan. Written informed consent was obtained from the owners for the participation of their animals in this study.

## Author Contributions

NS and TS designed the research and wrote the manuscript. NS and MI performed the research and analyzed the data. All authors contributed to the article and approved the submitted version.

## Conflict of Interest

The authors declare that the research was conducted in the absence of any commercial or financial relationships that could be construed as a potential conflict of interest.

## Publisher’s Note

All claims expressed in this article are solely those of the authors and do not necessarily represent those of their affiliated organizations, or those of the publisher, the editors and the reviewers. Any product that may be evaluated in this article, or claim that may be made by its manufacturer, is not guaranteed or endorsed by the publisher.
