# Supplementary figures and images for: D1 Receptor Mediated Dopaminergic Neurotransmission Facilitates Remote Memory of Contextual Fear Conditioning
Source: Front Behav Neurosci. 2022 Feb 17;16:751053. doi: 10.3389/fnbeh.2022.751053 (PMC8925912; doi:10.3389/fnbeh.2022.751053)

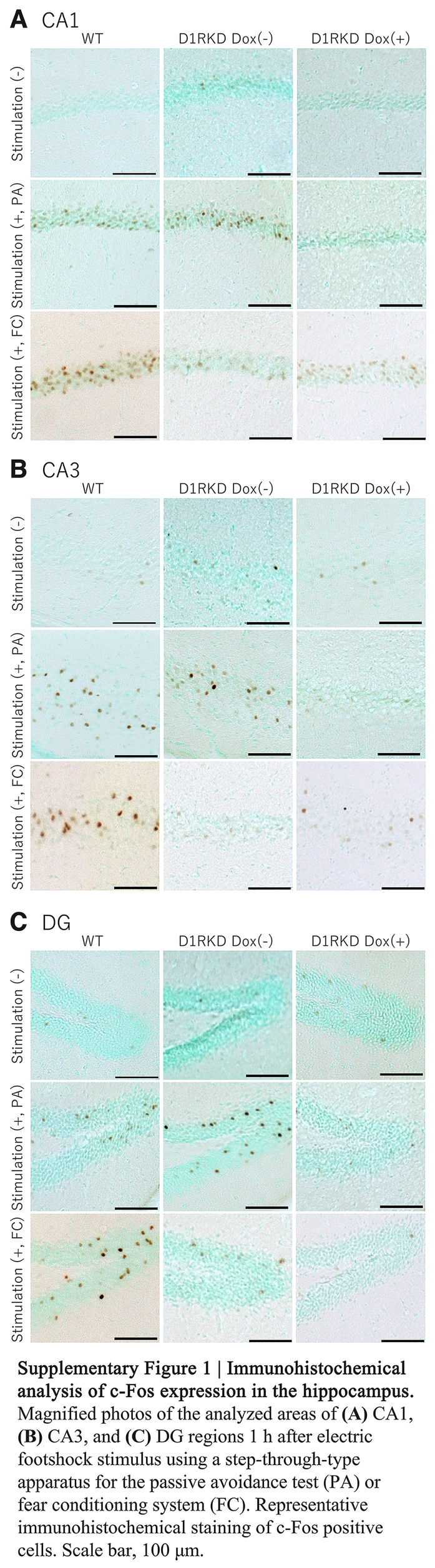

Supplement: Supplementary file 1 [file Image_1.tif]
